# A Pipe-Embeddable Impedance Sensor for Monitoring Water Leaks in Distribution Networks: Design and Validation

**DOI:** 10.3390/s23063117

**Published:** 2023-03-14

**Authors:** Danilo Aparecido Carnevale Castillo, Marco Carminati

**Affiliations:** 1 Dipartimento di Elettronica Informazione e Bioingegneria, Politecnico di Milano, 20133 Milano, Italy; 2 Istituto Nazionale di Fisica Nucleare (INFN), Sezione di Milano, 20133 Milano, Italy

**Keywords:** water leakage detection, impedimetric sensing, impedance spectroscopy

## Abstract

Water leakage is one of main problems of distribution infrastructures, reaching unacceptable peaks of 50% of water lost in old networks in several countries. In order to address this challenge, we present an impedance sensor able to detect small water leaks (below 1 L of released volume). The combination of real-time sensing and such a sensitivity allows for early warning and fast response. It relies on a set of robust longitudinal electrodes applied on the external surface of the pipe. The presence of water in the surrounding medium alters its impedance in a detectable way. We report detailed numerical simulations for the optimization of electrode geometry and sensing frequency (2 MHz), as well as the successful experimental proof in the laboratory of this approach for a pipe length of 45 cm. Moreover, we experimentally tested the dependence of the detected signal on the leak volume, temperature, and morphology of the soil. Finally, differential sensing is proposed and validated as a solution to reject drifts and spurious impedance variations due to environmental effects.

## 1. Introduction

Despite being a ubiquitous planet resource, less than 1% of water is available for human use. In a context of growing worldwide population, such a scarcity demands an adequate management of the full cycle of water, be it for domestic usage or for agriculture. According to the global water footprint estimations [1], the latter accounts for more than 90% of the total consumption, whereas the former encompasses only 4% of it. Therefore, water loss during transport is a widespread concern of utmost relevance that motivates the development of multiple procedures for pinpointing leakage sources within pipelined distribution networks [2,3].

Some approaches rely on first unravelling the leak occurrence by monitoring a dataset collected via dedicated sensors in specified nodes, e.g., abrupt flowrates and pressure discrepancies [4,5], to be further analyzed by a central control unit. Once an alarm has been triggered, subsequent steps are then followed for dynamically investigating, with better precision, the defective segment of the buried pipe. A customary approach is resorting to proficient technicians capable of handling non-invasive geophones on the ground and quantifying acoustic abnormalities throughout the topography. Similarly, infrared thermography [6], ground-penetrating radars [7], and sensors detecting vibration signatures [8] have also been proposed, aiming at the same objective. 

Another class of detectors, with higher sensitivity and potential for better spatial accuracy, incorporates more intrusive means that need access points to introduce sensors, as exemplified by noise correlators connected to the water system through fire hydrants [9]. In spite of some endeavors to miniaturize these devices, such as with MEMS hydrophones [10] and accelerometers [11], the necessity for a direct contact with the fluid or the pipe, respectively, remains unavoidable.

Research is focusing on developing non-invasive prognostic solutions rather than a posteriori diagnostic ones, thus identifying hydraulic leaks as early as possible while concurrently preventing severe network deterioration. For instance, optical fibers are unrolled along pipelines or coiled around pipes to locally detect water leaks as result of a change in the temperature or the dielectric properties of the fiber [12,13]. Electrical conductors can be drawn as well along pipes to perform time-domain reflectometry [14]. The main limitations of these approaches are the limited selectivity (due to cross-sensitivity to temperature and other effects) and the cost and complexity of the readout instrumentation. Impedance, instead, can be easily measured. For instance, impedance sensing was proposed for surface electrical resistance tomography [15] to map leaks. Impedance can be measured between electrodes running inside an outer insulating foam shell coating the pipe, as in the commercial system Detect by LOGSTOR. Unfortunately, the significant cost of manufacturing these supplementary layers narrows their application predominantly to the oil and gas industry.

We recently proposed a simple approach, requiring minimal modifications to the pipe, based on the application of longitudinal and parallel strip electrodes on insulating pipes. When water leaks, the electrical properties of the surrounding medium change and give rise to a detectable variation in impedance. The sensitive length corresponds to the length of the electrodes. Electrodes can be applied on pipes already installed, in specific segments where leakage risk is higher, hence simplifying their installation. The starting point of this work was a very preliminary and rudimentary simulation showing the feasibility of the impedimetric approach [16]. Interestingly, a similar approach has been proposed for power cables [17], as well as for indoor leak monitoring with dielectric tape [18].

**Figure 1:**
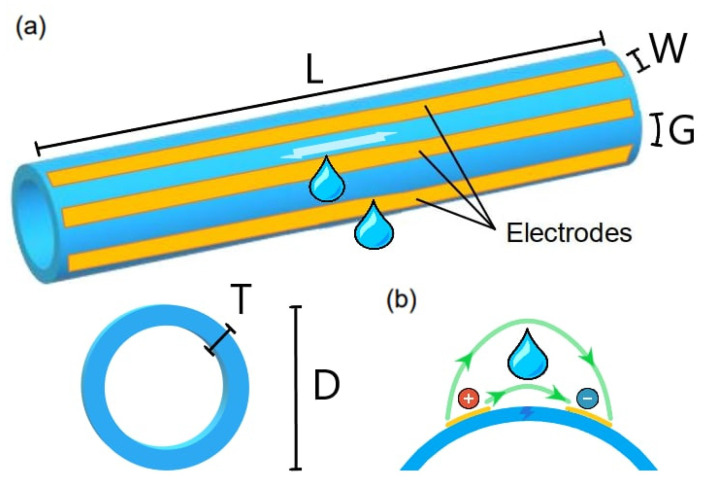
(**a**) Sensor’s geometrical parameters. (**b**) The electric field (in green) set between adjacent electrodes senses any alteration in the outer medium electrical properties, e.g., due to a water leak.

In this paper, we report refined numerical simulations and an extensive experimental characterization of this sensor concept. The reference geometry is shown in Figure 1. Electrodes have a longitudinal length L and width W and are separated by a gap G. They are applied on a PVC pipe of external diameter D = 200 mm and thickness T = 8.8 mm. For the static tests in the laboratory here reported, L was set to 45 cm for fitting in boxes of 50 cm length. The optimal sizing of G and W, which are the key parameters setting the volumetric sensitivity and the sensing frequency, is discussed in the next section. Their values can be adapted to different diameters of pipes, offering versatility. In an interdigitated configuration, where fringing fields dominate, the value of W impacts on the interface capacitance, while G sets the ionic resistance value of the fluid and the sensitive volume [19].

Electrodes were fabricated in copper, cut from commercial copper adhesive tape. In order to avoid their rapid oxidation when in contact with wet soil, they were passivated by a thin (50 μm) layer of Kapton^®^. These materials were not randomly chosen, once they are routinely employed for the manufacturing of flexible printed circuit boards (PCB) [20], thus representing an option for industrial fabrication of such strips of electrodes ready to be glued onto insulating pipes [21]. In the case of conductive pipes, a double-sided insulation is needed. Impedance spectra were acquired with a precision LCR meter (E4980a by Agilent, Santa Clara, CA, USA), setting a stimulation voltage of 1 V and an averaging time of 1 s.

## 2. Simulations

### 2.1. Enhanced Model

**Figure 2:**
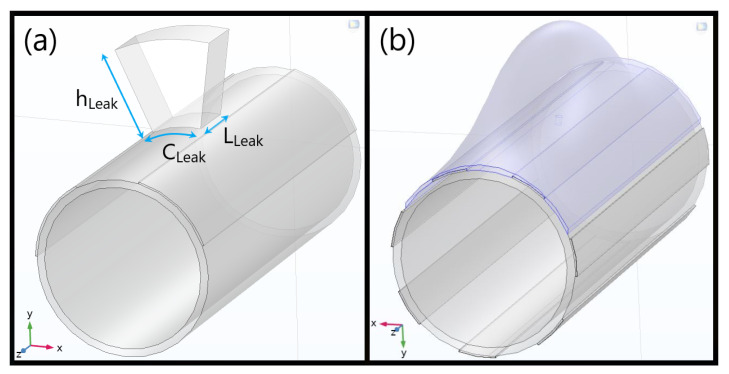
(**a**) Initial model of the leak geometry over a single pair of non-insulated copper electrodes, utilized for refining the gap (G) and width (W); (**b**) Final interdigitated structure adjusted after a few preliminary validation tests and exhibited in advance. An alternative 2 L Gaussian water loss profile is highlighted in blue with an amplitude concentric to a parameterized hole on top, as implemented in the real prototype and better shown in Figure 3a. This model includes both the 50 µm Kapton^®^ tape and the ionic double layer enfolding the electrodes.

The first step was to define a realistic geometry for the simulation domain. It was improved with respect to [16], where the wet volume was assumed to be a simple 10 cm^3^ cube placed on the electrodes. Initially, a seepage profile was tested (Figure 2a) by extruding a bidimensional circular sector along a length *L_Leak_* matching the arc segment *C_Leak_* (hence *L_Leak_* = *C_Leak_*) and later separating it into distinct material layers, with a similar approach for the 45 cm-long electrode pair at first. In specific, *C_Leak_* is the product between the angular aperture (*θ_Leak_*) and radius (*ρ_Leak_*), this one being the pipe radius (*ρ_Pipe_*), summed with the exposed Cu electrodes’ height (*h_Elect_*).

**Figure 3:**
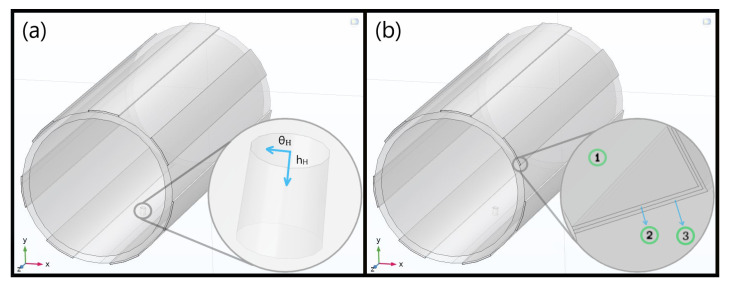
(**a**) Detail on conduit hole used to inject the leak in the real scenario, *θ_H_* being the angular coordinate and *h_H_* its own height variable (spanning up to the pipe thickness), both imposed to model its corresponding surfaces. Moreover, *θ_0,hole_* refers to the center of the inner aperture. (**b**) Passivation with 50 μm Kapton^®^ tape (2) and ionic double layer (3) on top of a 2 mm Cu electrode (1).

Such a model was still far from reality and, thus, a Gaussian surface as shown in Figure 2b was chosen and parametrized to simply describe water diffusion in the medium. Specifically, a conformal mapping departing from cylindrical coordinates (*ρ_G_, θ_G_, z_G_*) was defined to obtain a bidimensional Gaussian surface with an amplitude *A_G_*, bound by a minimum height limit equal to *ρ_0,G_* and centered at (*z_0,G_, θ_0,hole_*), i.e., the pipe longitudinal and hole middles, with associated standard deviations (*σ_θG_, σ_ZG_*) for (*θ_G_, z_G_*), respectively. Moreover, please note that the “G” subscripts for such variables stand for “Gaussian”, not for the gap. Finally, the leak lateral surfaces, the orifice, the redesigned electrodes, and their insulation tape and ionic double layers all underwent a similar path, some being shown in Figure 3. As a parameterization instance, the equations for the Gaussian-alike surface emulating the top leakage portion are presented in Table 1.

Concerning materials, PVC was set for the pipe, copper for the electrodes, and polyimide for the passivation. The thickness of the electrodes *h_Elect_* was set to 2 mm, much thicker than the actual one (~18 µm), to avoid excessive mesh details and memory use, without any drawback in the sensitivity estimation.

### 2.2. Leakage Spatial Detectability

In both previous models, the associated outflowing water volume is consistently controlled by the system parameters. In Figure 2a, the angular sector beneath the leak zone (*θ_Leak_*, in rad) has an implicit expression binding it to the volumetric loss magnitude and to *h_Leak_*:

$$V_{Leak}=L_{Leak}\cdot \pi \cdot \left[{\left(h_{Leak}+\rho_{Leak}\right)}^{2}-\rho_{Leak}^{2}\right]\cdot \left(\frac{\theta_{Leak}}{2\pi }\right),L_{Leak}=C_{Leak}=\theta_{Leak}\cdot \rho_{Leak}$$

(1) $$∴\theta_{Leak}^{2}=\frac{{2\cdot V}_{Leak}}{\rho_{Leak}\cdot (h_{Leak}^{2}+2\cdot h_{Leak}\cdot \rho_{Leak})}$$

Similarly, the leakage volume in Figure 2b (*V_G_*) can be coherently approximated by (2), which is solely an estimative not only because [*θ_G_, z_G_*] ∊ ℝ × ℝ is a finite real interval but also since the laterally imposed surfaces limiting the top one for properly confining a region in ℝ^3^ are not simple sharp edges. Nonetheless, the volume gathered outside them becomes negligible for spatial boundaries much greater than Gaussian standard deviations (*σ_θG_, σ_ZG_*) like the ones imposed, thus justifying the expression’s usage. Finally, the arc-segment length *C_θ_* drawn by *θ_G_* equals *ρ_0,G_·θ_G_*; hence, *dC_θ_* = *ρ_0,G_·dθ_G_*, *σ_Cθ_* = *ρ_0,G_·σ_θG_*, and:

$$f\left(z_{G},C_{\theta }\right)=A_{G}\cdot \exp\left\{-\left[\frac{{\left(z_{G}-z_{0,G}\right)}^{2}}{2\cdot \sigma_{z_{G}}^{2}}+\frac{{\rho_{0,G}^{2}\left(\theta_{G}-\theta_{0,hole}\right)}^{2}}{2\cdot {\left(\rho_{0,G}\sigma_{\theta_{G}}\right)}^{2}}\right]\right\}=f\left(z_{G},\theta_{G}\right)$$

(2) $$∴V_{G}\sim \int_{-\infty }^{\infty }\int_{-\infty }^{\infty }f\left(z_{G},C_{\theta }\right)dz_{G}dC_{\theta }=2\pi A_{G}\sigma_{z_{G}}\rho_{0,G}\sigma_{\theta_{G}}$$

*N.B*.: *C_θ_* → *± ∞* has no proper physical meaning in this case; by construction, the Gaussian surface will just keep revolving around the cylinder for *θ_G_* > *θ_0,hole_ +* π or *θ_G_* < *θ_0,hole_* - π.

In order to ascertain which variable in (1) among *θ_Leak_* and *h_Leak_* has a prevailing impact on the soil absolute impedance variation, henceforth denominated Δ|Z_Soil_(jω)|, for a target *V_Leak_*, *θ_Leak_* was fixed to encompass a respective 5 cm arc-segment and so was *L_Leak_*. Table 2 sums up how the self-adaptable *h_Leak_*, as a function of *V_Leak_*, impinges on Z_Soil_(jω) at 1 kHz for an electrode pair with a corresponding gap and width of 5 and 10 cm, with a dry soil conductivity (*σ_Soil_*) of 1 μS/cm and relative permittivity ε_r,Soil_ = 10, whereas ε_r,Leak_ = 80.

Although Z_Soil_(jω) respects an expected hyperbolic demeanor for a steady *V_Leak_* when facing min{*σ_Leak_*} up to max{*σ_Leak_*} and naturally diminishes for a constant *σ_Leak_* with increasing *V_Leak_*, the outcomes support that a greater leak volume spreading away from the sensor seems to saturate its sensitivity. Despite some inherent counterintuitiveness, the relative variation between the dry baseline (BL) and an entirely wet soil is almost constant regardless of the greater *h_Leak_* dimensions, since most of the coplanar electrodes electric field is foreseen to be gathered within a height roughly equivalent to their gap [22], an inference that could be naturally extended to the quasi-coplanar case investigated. Therefore, no noticeable impedance fluctuation would be presumed to occur a priori once the *h_Leak_* overcomes the 5 cm gap; ergo, this is in compliance with the simulated scenery.

On the contrary, a steady *h_Leak_* of 10 cm with modifiable base dimensions via *θ_Leak_* and *L_Leak_* for setting a desired *V_Leak_* was also emulated, and it ratified how raising *h_Leak_* for a constant base extension has, indeed, a weaker influence on the system responsiveness than adopting a laterally disseminating water loss with a steady height. As a result, all ensuing simulations adopted the latter procedure, e.g., the Gaussian volume from (2), whose amplitude *A_G_* was set to 10 cm with only its standard deviations varying accordingly.

### 2.3. Electrodes Size Optimization

A heuristic approach had to be pursued for defining the best G and W parameters capable of maximizing the detector sensitivity to a leak spreading in between a pair of exposed copper electrodes. This method became a more feasible approach than the Schwartz–Christoffel conformal mapping, a holomorphic function transformation that conserves local angles and consistently estimates the electric field strength for perfectly coplanar electrodes as in the microfluidic domain [22]. Therefore, the CAD model from Figure 2a was hereby embraced for inspecting the leakage sensitivity enhancement when modulating such dimensions. The centers of the outflowing water and the metal strips gap were also suitably parametrized to overlap autonomously regardless of G and W.

Firstly, some narrowed yet feasible gap and width extensions for an individual electrode couple were scrutinized to map their influence onto the ability to perceive a local impedance alteration due to the accumulated moisture. In specific, the (G, W) coordinates ranged by equidistant 25 mm intervals in the [25, 100] × [25, 100] Cartesian plane in mm, being later refined to seek an optimal (G, W) that could not only fit an entire interdigital structure but also economize the consumed materials. Analogously to before, the analysis was thereby built upon alternating the leak lateral dimensions and carrying on with the heretofore imposed readout frequency of 1 kHz, at which point the exposed electrodes present a dominantly resistive behavior. As forecasted, this value was further compelled to increase due to the Kapton^®^ tape entrance capacitance further lidding the electrodes. 

**Figure 4:**
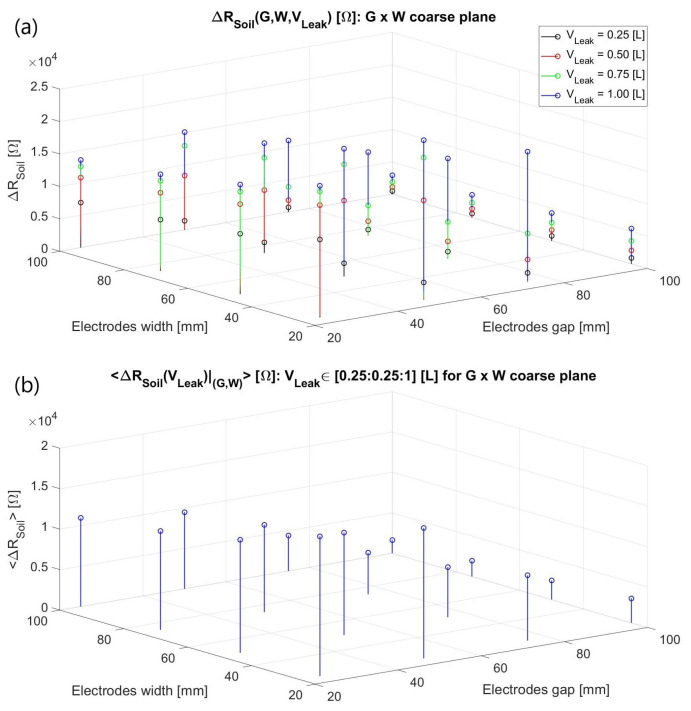
(**a**) Three-dimensional discrete function ΔR_Soil_ (G, W, V_Leak_): ℝ^3^ → ℝ elucidating the Δ|Z_Soil_(jω)| dependence on the metal strips’ gap and width and the water loss volume at 1 kHz for an arid-to-moist terrain with a nearly resistive behavior; (**b**) The preceding results were averaged for the investigated *V_Leak_* volumes at each (G, W) to circumvent potentially misleading conclusions.

As exhibited in Figure 4a, the verified ΔZ_Soil_(jω) for constant (G, W) are consistently more dependent on *V_Leak_* for a predefined *h_Leak_* of 10 cm rather than being quasi-steady with respect to it as in the radial water dispersal antecedent condition. Moreover, four distinct loss volumes, with *σ_Leak_* once more spanning from 1 to 500 µS/cm, were examined due to the modelled infiltration geometry. On one hand, a higher impedance variation happens when the leak extension entirely encompasses a minor gap and moderately stretches over the metal surfaces compared to when it leaves them untouched. For instance, a W of 2.5 cm using a 0.25 L *V_Leak_* (*L_Leak_* = *C_Leak_* ~ 4.1 cm) has Δ|Z_Soil_(jω)|_(G = 50 mm)_ < Δ|Z_Soil_(jω)|_(G = 25 mm)_.

Conversely, Δ|Z_Soil_(jω)|_(G = 50 mm)_ > Δ|Z_Soil_(jω)|_(G = 25 mm)_ was obtained in the 1 L loss scenario despite its *L_Leak_* of around 8.2 cm fully covering both gaps, completely lidding the tinier gap electrodes and about half the more separated ones. Consequently, a slightly greater distance between them seems to entail a broader monitorable soil domain more prone to testify to the humidity presence in the two situations where the metal strips are somehow reasonably covered by the leakage. Both reasonings might also justify why a greater ∂(Δ|Z_Soil_(jω)|)/∂*V_Leak_* occurs for G = 5 cm rather than for the shorter one. Similarly, reducing the width for the steady gaps enhances the sensitivities in the studied range probably due to better focusing of the electric field majority towards the leak zone.

**Figure 5:**
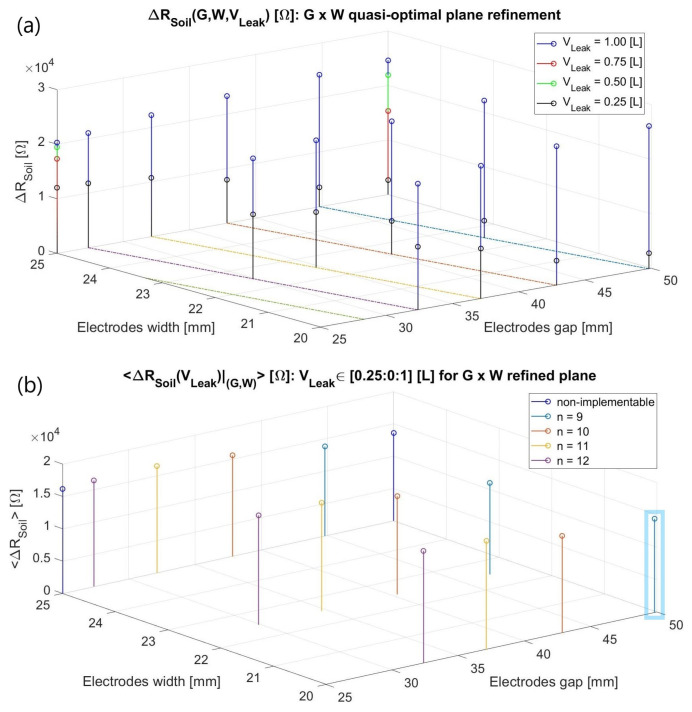
(**a**) Heuristically optimal G × W refinement for doable dimensions according to (3). A color pattern was established for *n* ranging from 13 (greenish) to 9 (blueish); (**b**) Averaged outcomes for implementable extensions, the final selected (G, W) = (5, 2) cm being highlighted in blue.

Finally, averaging the obtained results (Figure 4b) emerged as an endeavor to more consistently predict the system’s resolution of a leak detection due to the latter intricate volumetric influence even for a simplified portrayal. Subsequently, a narrowed G × W window of [25, 50] × [20, 25] in mm was refined and inspected (Figure 5), departing from the above chart’s best results. In this case, only two leakage volumes were tested (0.25 and 1 L), exclusively for (G, W) values implementable in the final interdigitated structure, i.e., capable of encircling the 200 mm-diameter pipe by a natural number of repetitions:

(3) $$G+W=\frac{2\pi 100}{n},\;n∊N^{*}$$

The *n* variable in (3) ranged from thirteen to nine electrodes, being represented by colored lines in the G × W refined plane (Figure 5a). Once Δ|Z_Soil_(jω)| was averaged at 1 kHz for such ameliorated (G, W) coordinates (Figure 5b), no huge shift was attained. Therefore, the final design verdict up to this point was to select the largest gap and lowest width within them, (G, W) = (50, 20) mm, for the smallest amount of interdigitated repetitions to be achieved so to save the manufacturing materials and reduce the sensor cost.

## 3. Experimental Results

### 3.1. Preliminary Measurements

#### 3.1.1. Single Electrode Pair

**Figure 6:**
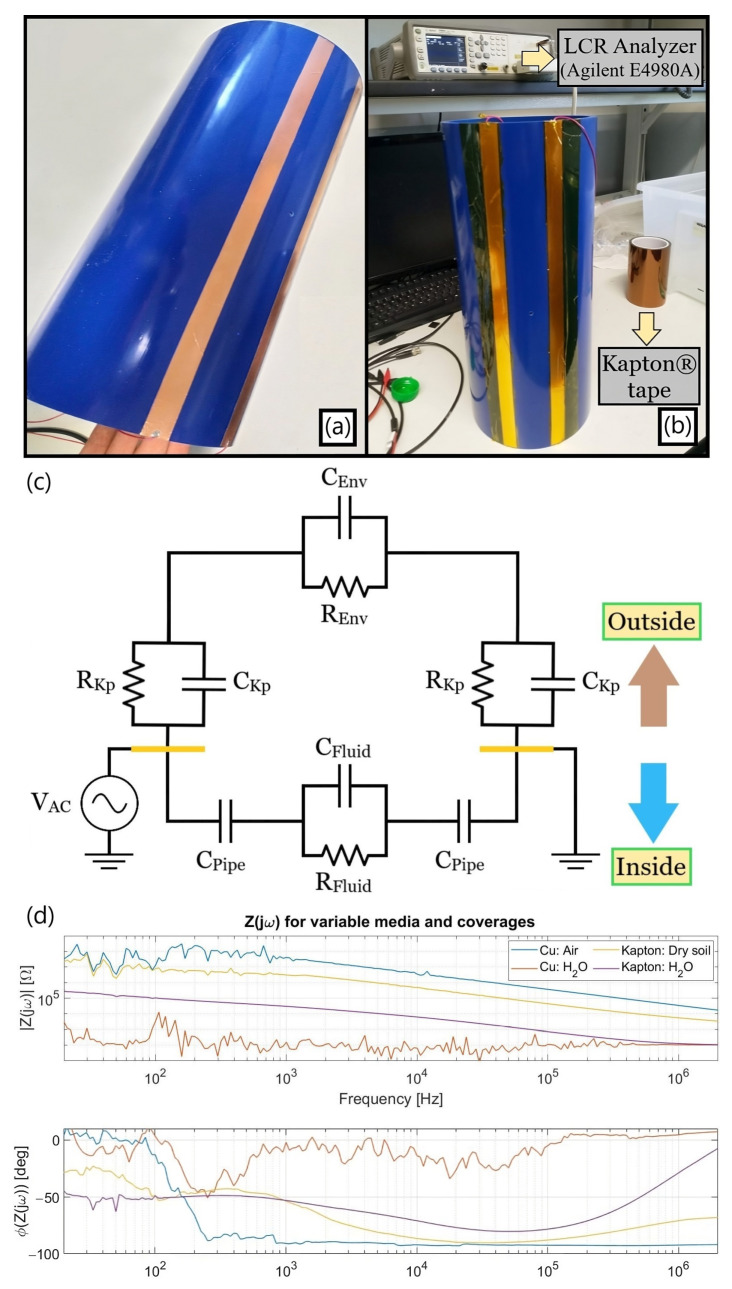
(**a**) Exposed copper strips with the heuristically optimal dimensions (G, W) = (50, 20) [mm, mm]; (**b**) Kapton^®^-covered electrodes and the LCR instrument capable of measuring up to 2 MHz; (**c**) First improved electrical equivalent model, further revisited after some adjustments in the empirical setup; (**d**) Initial impedance spectra set gauged with the LCR instrument when air filled the pipe.

The initial physical prototype relied on a 45 cm-long PVC tube (Fitt Bluforce) with the previously detailed geometrical parameters, the optimized electrodes being implemented using copper and Kapton^®^ tapes as in Figure 6a,b. Its first improved electrical equivalent circuit (Figure 6c) sums up both the outer and inner main components for a pair of electrodes diagrammed in yellow and biased through an AC voltage source a priori, the copper’s resistive and capacitive effects being neglected due to its elevated conductivity and presumed incapacity to be polarized. The scheme exhibits the C_Env_ and R_Env_ elements from the distinct media encircling the conduit as well as the impact of the metal strips’ insulation, in which C_Kp_ stands for their insulating barrier whereas the finite R_Kp_ is related to the Kapton^®^ non-null current flow at the quasi-DC range. Their holistic aftermath can be testified to by the Z(jω) spectra gauged with an LCR apparatus for air filling the pipe (Figure 6d), with exposed and later insulated strips facing distinct outer media when the sensor region was fully enfolded by them but only partially covering the remaining tube extension (except for air).

**Figure 7:**
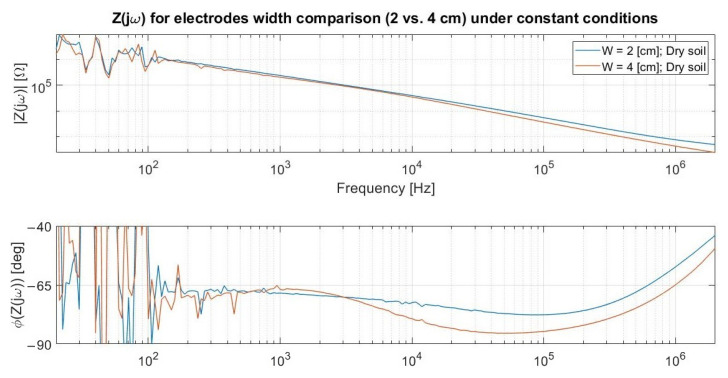
The LCR outcomes confirm that wider electrodes increase C_Kp_ but do not grant a noteworthy variation on its zero preceding the resistive plateau due to a concomitant R_Soil_ reduction.

Among the primary conclusions from the above charts at Figure 6d are:

(1) *Exposed electrodes outcomes:* The outer air environment is sensed by the non-insulated metal strips as an almost ideal capacitance from the intermediate frequency range onwards due to its poor electrical conductivity. On the other hand, the water ionic composition leads to a quasi-resistive comportment. The entrance capacitive element C_Kp_ introduced by the polyimide coverage has an impact of up to 1 MHz when comparing the two aqueous scenarios, above which the impedance converges to the R_H20_ resistive plateau in both cases. Finally, no exposed copper electrodes test with an encircling dry soil was performed hitherto because it would not consist of the final target design.
(2) *Insulation issue:* The shortening process for the C_Kp_ element in the dry soil scenario can be verified at the yellow phase plot curve at around the same frequency order of magnitude as in the aqueous one. However, its inferior ∂(Φ_Soil_)/∂f leads to a lower phase peaking and implies that R_Soil_ tends to vanish within |Z(jω)| due to the proximity between the C_Kp_ zero singularity and a preexistent higher-frequency pole related to C_Soil_ and R_Soil_. Although imposing a readout frequency (f_R_) above the original 1 kHz was a foreseen requirement due to the C_Kp_’s existence along with a real ground conductivity diminishing the predicted R_Soil_ from being about one hundred times superior to the initially surmised 1 µS/cm as later estimated, a complementary alternative had to be devised for trying to reduce the aforesaid zero so as to still enable tracking of the R_Soil_ plateau.
(3) *Insulation alternative:* The sensor width was doubled from 20 to 40 mm as an endeavor to reduce f_R_ by enlarging C_Kp_ and empirically derive such a widening impact departing from the originally quasi-optimal 2 cm dimension. As can be ascertained in Figure 7, not only did the C_Kp_ increase but also R_Soil_ diminished, since the former depends linearly at a first order on the electrodes’ area whereas the latter does so hyperbolically. Nonetheless, both their intricate relation with the residual geometrical parameters and the electric field spatial distribution still culminated in a slight alteration at the zero singularity.

Despite stepping out of the refined optimal zone entailed by the analysis in Section 2.3, (G, W) = (50, 40) [mm, mm] still grants a considerable average sensitivity since it is placed reasonably near the heuristically optimal coordinates. The widened electrodes were maintained to the forthcoming interdigitated structure since, in terms of readout capability, a larger current is achieved at the MHz range for a constant voltage biasing. Nevertheless, a future distinct readout topology might be bounded by its impedance resolution, e.g., when dealing with longer strips, in which case this topic ought to be carefully reexamined. 

**Figure 8:**
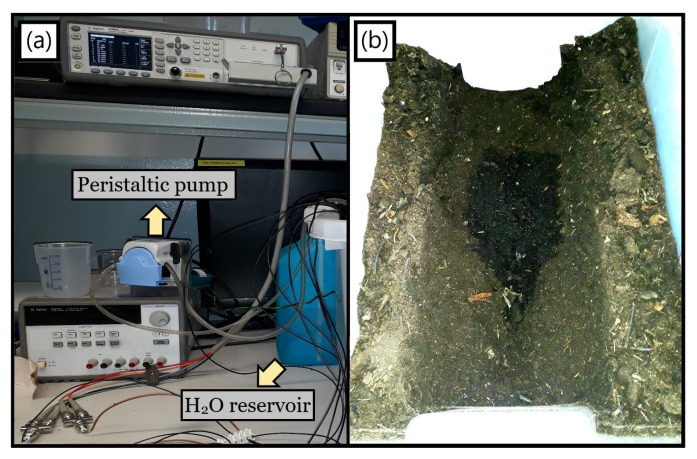
(**a**) Full setup for emulating a monitored leakage; (**b**) Real water loss photograph taken from above the rectangular plastic box initially filled with dry soil.

Concerning the method for assaying real leakages, a peristaltic pump was utilized to drain water from a reservoir (Figure 8a) and control the outflowing volume, guiding it via a hose connected to a tiny hole drilled in between the single electrodes gap. Such a procedure was imposed for all upcoming detection tests related to a supervised water loss dissemination within a commercial soil positioned inside a plastic box capable of fitting the setup, a well-defined leakage picture being displayed by the darker zone in Figure 8b.

Finally, the following topic demonstrates how a minimum amount of ground about three times the imposed electrodes gap must necessarily encompass the sensor so as not to inadequately culminate in a jeopardized |Z_Soil_(jω)| spectrum, a condition testified to only after the early trials exposed it. Therefore, despite some tests with a single strips pair sensing a pumped leak and so indeed attesting to the system’s feasibility for shallower soil heights, they were not entirely consistent due to the effect in Section 3.1.2 and are not hereby exhibited. 

However, once the procedures for circumventing such spurious influences were discovered, they were then applied for obtaining a spectra and leakage sensitivity comparison between corrected and compromised physical setups using the interdigitated structure as shown in Section 3.2. Furthermore, some modifications also had to be imposed on the equivalent circuit from Figure 6d so that the simulated F.E.M. electrical parameters and corresponding spectra could more coherently approach the rectified Z_Soil_(jω). 

#### 3.1.2. Effect of Soil Thickness

**Figure 9:**
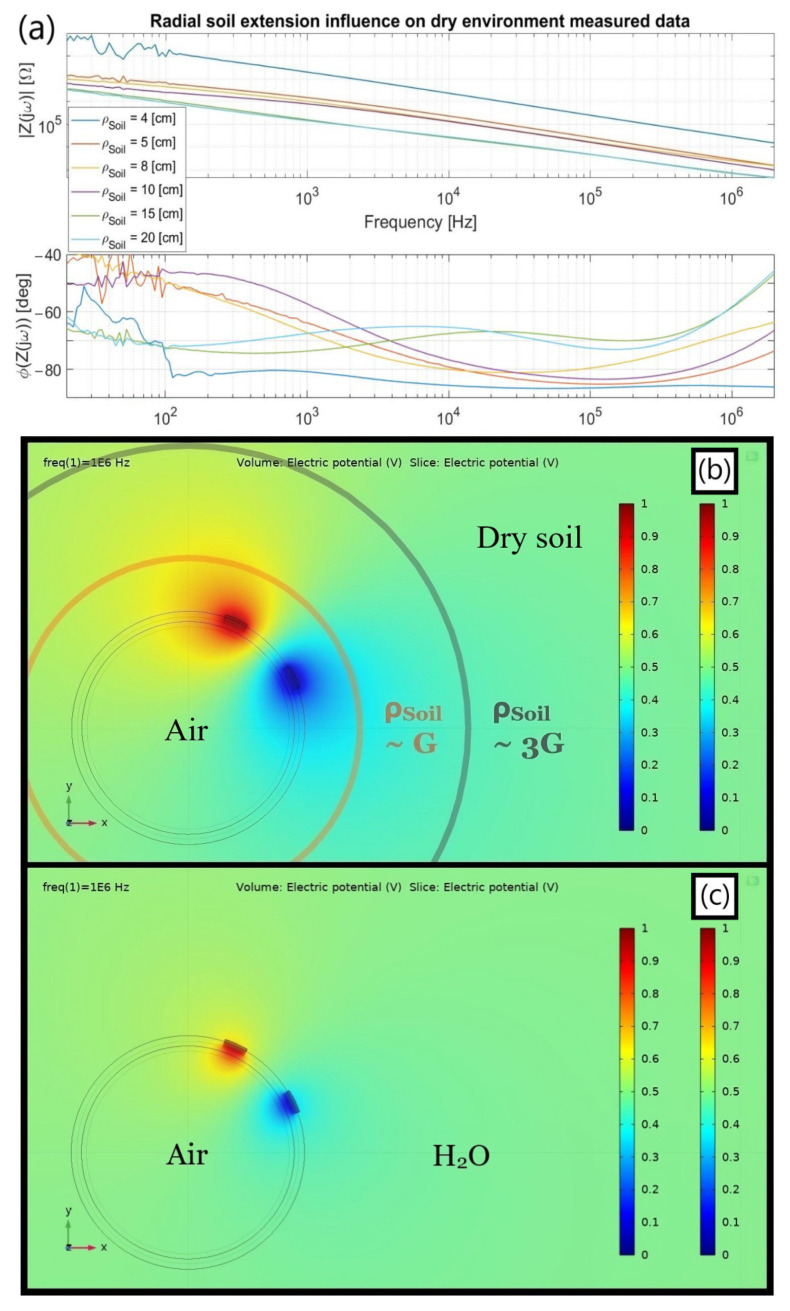
(**a**) Z_Soil_(jω) spectra for an electrode pair with (G, W) = (5, 2) cm on a pipe filled with air when facing variable ground heights; (**b**) F.E.M simulation for the electric field dispersion at 1 MHz along the xy plane using a soil medium outside, with σ_Air_ = 0 µS/cm and ε_R,Air_ = 1 whereas σ_Soil_ = 100 µS/cm and ε_R,Soil_ = 10. (**c**) Same as (**b**) with water surrounding (σ_H2O_ = 1000 µS/cm and ε_R_,_H2O_ = 80).

Albeit a micrometric coplanar electrode pair experiences a millimetric liquid drop as an “ocean”, not adopting a properly scaled terrain in accordance with the involved dimensions entails misleading results as witnessed in Figure 9a by the noticeable Z(jω) fluctuations when altering the soil thickness around the electrodes. Such a plot underlines the obligation of setting a minimal radial dimension for the ground around the tube to be considered virtually infinite, specifically about three times the gap size since G = 5 cm and *ρ_Soil,min_* ~ 15 cm allows achieving a steadier frequency spectrum. This effect is hypothesized to be intrinsically bonded to the electric field spatial distribution in between the electrodes, depending on the amount of material enveloping the latter as shown in Figure 9b.

As previously mentioned, the quasi-coplanar sensor’s radial responsiveness is associated with the gap (G) between the electrodes. Nevertheless, its electric field attains a vaster projection (Figure 9b) that must not be disregarded for circumventing the previous incorrect aftermath. In specific, a shallower terrain radius encircling the pipe might unavoidably compress the electric field to a narrower transversal area (A’), hence concomitantly impacting R_Env_ and C_Env_, namely, R_Soil_ and C_Soil_. At a first order, these parameters are, respectively, proportional to 1/A’ and A’, so reducing such a surface by using a ground of little depth surrounded as well by an insulating plastic box on the bottom might be the main accountable justification for lifting the whole |Z_Soil_(jω)| spectrum up.

Moreover, another noticeable aspect regards how no homogeneous impedance can be sensed apparently for the terrain, only an averaged one within a specific arrangement. For instance, the data in Figure 9a was collected throughout two days using distinct terrain packages, the first of which with *ρ_Soil_* of 4 and 8 cm. As a result, a slight unconformity befell for an 8 cm soil height beneath the electrode pair compared to the other values after rearranging the terrain, as can be inspected by the phase spectrum that should, a priori, have been obtained in between those from *ρ_Soil_* equal to 5 and 10 cm. 

The foregoing inference of imposing *ρ_Soil_* > 3 G was extended to the ensuing setups from the early moment of its discovery, even though fully covering the pipe with soil became a further defying challenge to overcome specially for the interdigitated structure. The epiphany that triggered such a modification ultimately arose when confronting both simulation and real measurements for the leak occurrences in topic 3.2 since the electrical parameters for matching both were unfeasible and inconsistent at first. 

In addition, an initial *ρ_Soil_* much greater than the G parameter was not set right at the early stages since quasi-coinciding spectra were achieved when confronting the simulated and real case scenarios for water surrounding the insulated strips (Figure 6d). In particular, they were contiguous enough at the capacitive intermediate frequency range for an imposed aqueous height close to G. Nonetheless, the electromagnetic lines in this condition (Figure 9c) do not propagate as far as in the arid soil situation, therefore inducing a misleading conclusion that no influence whatsoever on the gauged spectrum occurs.

Finally, among the additional illations that can be inferred based on the propounded arguments for the presented chart with the Z(jω) variable spectra depending on the *ρ_Soil_* dimension and enlightened by the electrical equivalent model introduced at Figure 6c are:

(1) *Environmental elements disguise:* For the lowest *ρ_Soil_*, the C_Soil_ and R_Soil_ parallel connection seems to become virtually huge to the point that the inner capacitive branch formed by the series of C_Pipe_ and C_Air_ tends to dominate the outer vs. inner current dispute. As inspected in Figure 9a, an essentially steady −90° phase is achieved apart from the initially bouncy values, presumably due to a large R_Air_ soon shortened by C_Air_, hence setting an internal capacitive coupling behavior. As *ρ_Soil_* increases, such an inner dominance seems to be overtaken by the outer branch since R_Soil_ decreases and C_Soil_ enlarges to their expected values for an “infinite” outer medium radius, a priori being *ρ_Soil_* > 3 G. Consequently, the greater *ρ_Soil_*, the smaller the inner capacitive effect and the easier the R_Soil_ measurability.
(2) *Prototype correction:* Besides *ρ_Soil_*, the ongoing geometrical dependence also has a non-negligible influence even for the lateral plastic walls holding the soil altogether. Although not hereby shown for the sake of simplicity, positioning the tube longitudinal axis parallel to the shorter or longer box sides slightly influences the outcomes, the former having more consistent results since a larger terrain amount radially enfolds the electrodes.
(3) *C_Kp_* vs. *Constant Phase Element (CPE):* the intermediate and low frequency ranges in Figure 9a were not focused due to some intrinsic non-linearities beyond the simplified proposed circuit scope. For instance, there is a zero-pole cancellation tendency at intermediate frequencies that evolves to a CPE demeanor in the interdigitated structure when both impose correct *ρ_Soil_* values. As will be shown, the latter replaces C_Kp_ in a refined equivalent model and exists primarily due to a thin ionic double layer formed on top of the polyimide tape that affects not only the access capacitance but also the local conductivity around it.

### 3.2. Interdigitated Structure 

#### 3.2.1. Single-Ended Leakage Detection

**Figure 10:**
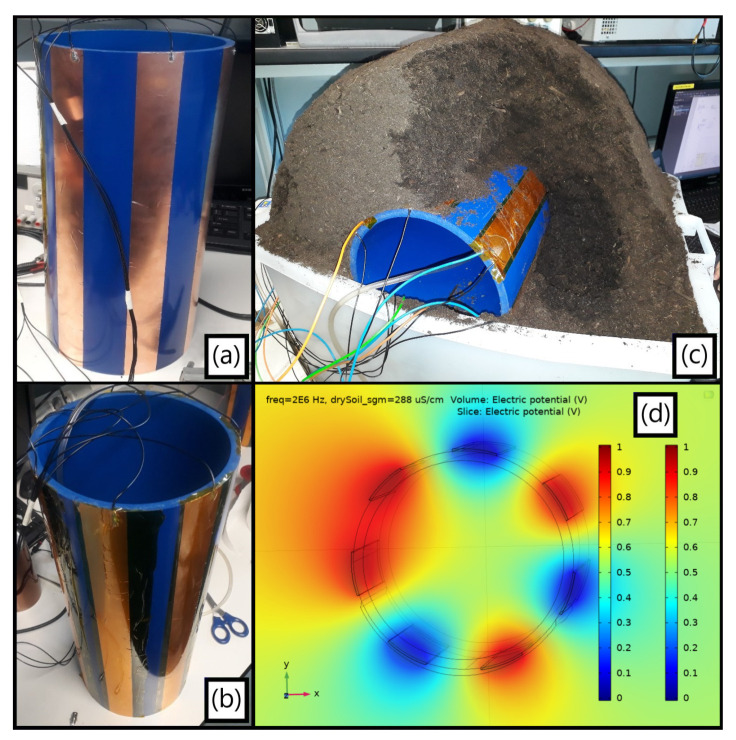
(**a**) Fully interdigitated sensor with seven embedded non-insulated electrodes relying on an optimized (G, W) = (5, 4) cm; (**b**) Idem, applying the Kapton^®^ tape; (**c**) Lateral leakage test final portrayal once the soil thickness was corrected; (**d**) F.E.M. electrical profile at 2 MHz for an encircling soil terrain and air inside the tube, with a Gaussian leak geometrical artifact visible on the bottom.

**Figure 11:**
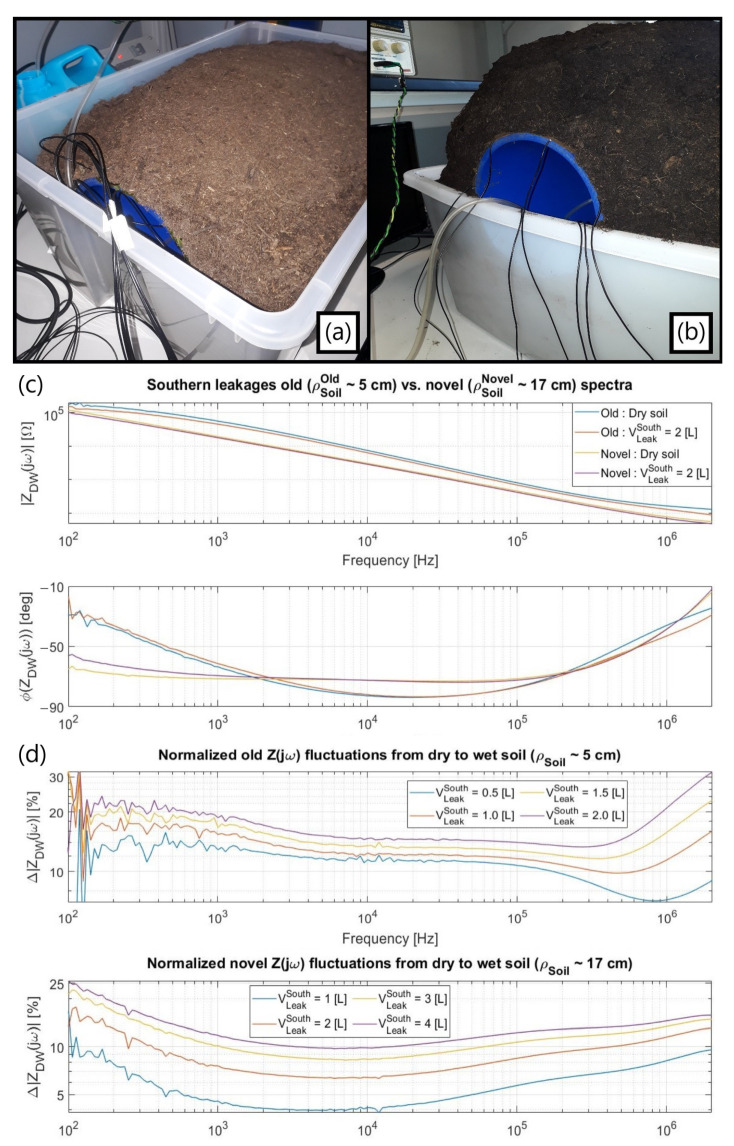
(**a**) First shallower terrain thickness of about 5 cm tested with the single-ended approach; (**b**) Deeper soil radius of around 17 cm, ensuring the *ρ_Soil_* > 3G principle from Section 3.1.2. Its darker color is caused by accumulated background humidity; (**c**) Comparison between the dry-to-wet impedance spectra, namely Z_DW_(jω), in both cases; (**d**) Absolute impedance variations for increasing water volumes dispersed within setups normalized by their dry baselines.

In the single-ended method, four electrodes from Figure 10b were biased at V_DD_ whereas the other three remained at a virtual ground for establishing the current readout (Figure 10d), the amount of soil around the setup being considerably enlarged (Figure 10c) to overcome misleading spectra results. A comparison between two situations with shallower (Figure 11a) and thicker (Figure 11b) terrain radii is exhibited along with their respective spectra when detecting a water loss flowing downwards (Figure 11c), with the distinct individual impedance variations for each scenario at 2 MHz presented at Table 3. 

Both setups demonstrated a good, normalized shift in both magnitude (Figure 11d) and phase along the entire analyzed spectra from distinct origins later inspected by refining the system electrical parameters in C O M S O L. The improper shallower setup showed larger sensitivities not only due to its more spatially concentrated electric field but also since the thicker terrain had its leak detection capability reduced from background moisture accumulated from previous tests. It is emphasized that the generic commercial soils used had also been verified not to follow a prompt drying process.

#### 3.2.2. Electrical Parameters

An estimate for the electrical parameters was sought via C O M S O L for each setup in Figure 11, specifically by refining the involved conductivities and relative permittivities until reaching consistent convergence between the real measured and simulated spectra. This approach was used both for the gauged dry baseline and the southwards-pumped leakage spectra, whose digital comparison was the 2 L Gaussian-alike leak model covering two electrodes (Figure 2b). Apart from the water loss zone, the domains with a major impact within [0.1, 2 10^3^] kHz were proven to be the encircling dry soil, the non-ideal insulating polyimide tape, and the ionic double layer (dL) formed on top of it. 

A priori, the dL existence in series to the Kapton^®^ explained why |Z_Soil_(jω)| was greater than expected at the [10, 100] kHz capacitive zone both for dry and wet soils, being first modelled as a fictitious 50 µm stratum (nanometric, in reality) fully covering the metal strips (Figure 3b) and used for extracting parameters relying on the shallower setup results (Figure 11a). Though most behavioral conclusions from this comparison between simulated and measured spectra can be extended for the thicker terrain situation (Figure 11b), a primary modelling adjustment had to be made for attaining more coherent parameters when dealing with the corrected setup. In specific, the initially simulated 50 µm ionic layer on top was removed whereas the bottom one (Kapton^®^, also 50 µm thick) was redefined to unify both layers and include a Constant Phase Element (CPE) behavior.

The pivotal influences derived from each topological portion are therefore subsequently exposed, with the best-fitting parameters from each case summed up in Table 4:

(1) *Initial double layer and Kapton^®^:* The same (σ_dL,Kp_)_dry_ conductivity was used for both equally sized layers in a dry terrain scenario and for electrodes not affected by the leak, strongly influencing the spectra below 1 kHz. However, the obtained double layer permittivity of (ε_r,dL_)_dry_ = 0.35 is not feasible since an even tinier one would be achieved for its nanometric real thickness, this being the main reason to impose a thicker soil when seeking for a better consistency. As for the local conductivity around their insulating material, it increases when electrodes are directly covered by wet soil, a fact impacting the low-frequency range and modelled through a higher double layer conductivity for the two strips covered by the leakage. Similarly, the water dissemination introduces a wider ionic concentration into the terrain, overcoming that of an arid soil, hence rising the local (ε_r,dL_)_wet_ for the metal strips sensing it and diminishing the magnitude plot at [10, 100] kHz due to a smaller |1/jωC_eq_|, with C_eq_^−1^ = C_Kp_^−1^ + C_dL_^−1^. Nonetheless, as occurred for (ε_r,dL_)_dry_, the best-fit cases once more converged to a physically incoherent value lower than the unit;
(2) *Unified ionic double layer and Kapton^®^ (CPE//R_eq_*): For simulating a CPE in the F.E.M. environment, an imaginary relative permittivity was imposed for the unified insulating layer, with (ε_dL+Kp_)_dry_ = α ε_Kp_ (jω)^−0.17^, α ∊ ℝ, and ε_Kp_ = 3. Such an expression did properly satisfy both the achieved phase at the spectra intermediate ranges and the magnitude-frequency-dependent slope. Intriguingly, the CPE permittivity module |ε_r,CPE_| also impinges on the ΔR_Soil_ variation above 1 MHz through the α factor instead of solely in the region at which a regular capacitive entrance element should dominate. Hence, (σ_Gnd_)_wet_ was not adequately pinpointed as in the antecedent numerical outcomes so that only an estimate is exhibited for the latter. Finally, the polyimide finite resistance dominates the equivalent one (R_eq_) and mostly impacts the low-frequency range, (σ_dL+Kp_)_dry_ being one order of magnitude smaller than in the incorrect setup and increasing anew in the presence of water.
(3) *Surrounding terrain and confined leak zone:* The dry terrain conductivity (σ_Gnd_)_dry_ impacts the spectra above 1 MHz and the zero singularity preceding the R_Soil_ plateau. Its permittivity directly influences the pole after the R_Soil_ zone, affecting mainly the verified phase around 1 MHz (LCR instrument limits the real detection up to 2 MHz). As for the wet terrain, increasing the confined 2 L Gaussian-alike infiltration conductivity indeed reduces the R_Soil_ plateau as foreseen. Regarding its permittivity, a higher C_Soil_ is attained when wet since ε_r,H2O_ > ε_r,Gnd_, so once R_Soil_ has been shortened by C_Soil_ at frequencies above the LCR bandwidth, a shrinkage in the magnitude spectrum ultimately occurs as a result.

As a matter of fact, a greater (σ_Gnd_)_dry_ was achieved in the enhanced setup both from correcting the effect from Section 3.1.2 but also due to the accumulated humidity from the anterior tests performed in the same reutilized terrain. Additionally, (ε_Gnd_)_wet_ = 60 for this condition remained a non-refined value within the leak region, although it could have been to better match the LCR gauged spectra around the MHz range.

#### 3.2.3. Refined Equivalent Circuit 

**Figure 12:**
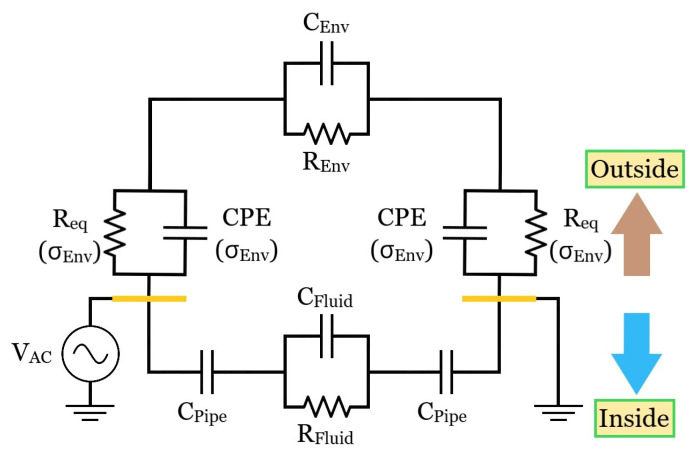
Refined equivalent circuit obtained via the thicker setup outcomes. Its yellow parts stand for three electrodes at virtual ground whereas the remaining four are at V_DD_. Furthermore, Req is the series between the polyimide and double layer resistances, the latter a function of environmental electrical conductivity (σ_Env_), whereas CPE(σ_Env_) represents the Constant Phase Element.

Relying on the antecedent outcomes after defining an adequate thick setup and extracting more accurate electrical parameters, an ameliorated topological description compared to Figure 6c was derived (Figure 12) and is presented along with its primordial expected and testified comportments when facing distinct surrounding media.

(1) *Outer air environment:* Due to the air’s poor electrical conductivity and permittivity, considerably large R_Air_ and C_Air_ are obtained for this external vicinity. As a result, given that R_eq_ also has a high impedance derived from the non-ideal insulating material, a reduced associated pole is predicted to occur so that an outer capacitive coupling is already reached from low frequencies. A similar coupling occurs in principle at the inner branch when air fills the conduit due to its tiny respective pole, presenting then a balanced impedance with respect to the outside. Conversely, a water filling is foreseen to entail a tendency for the internal impedance path to dominate the outer vs. inner parallel connection and ultimately reduce |Z(jω)| compared to the “empty” condition since R_H2O_ << R_Air_ and C_H2O_ >> C_Air_. Moreover, the inner branch pole would be pushed forward in the spectrum (ε_H2O_/σ_H2O_ << ε_Air_/σ_Air_), possibly culminating in a potentially trackable R_H2O_ high-frequency plateau.
(2) *Outer soil environment (shallow terrain)*: As advocated for in Section 3.1.2, the electric field between the metal strips seems to be unavoidably compressed to a narrower transversal area if a proper amount of soil surrounding the pipe is not imposed, therefore virtually increasing R_Soil_ and reducing C_Soil_ so that a considerable part of the |Z(jω)| spectrum is lifted up. As a result, the CPE effect attained for the single-ended interdigitated scheme is reduced to an almost ideal capacitive demeanor at the intermediate spectra range. Nonetheless, the impedance related to the entrance capacitance region is still reduced from the water leak impact on the double layer, i.e., C_eq_^−1^ = C_Kp_^−1^ + C_dL_^−1^ decreases due to a greater C_dL_.
(3) *Outer soil environment (thick terrain)*: Apart from ensuring more accurate results, the corrected R_Soil_ and C_Soil_ could be potentially jeopardized in terms of the leak detection by the inner branch when moving from air to water filling since the internal path could potentially compete with the outer branch for the electrodes’ current and compromise the measurement. However, some empirical outcomes performed for the pipe filled with water and surrounded by soil still endorsed the leakage-sensing feasibility. A viable explanation is that the inner water-related pole is likely pushed forward beyond the LCR meter limit of 2 MHz resolution, hence not compromising the Δ|Z(jω)| variation at the R_Soil_ spectrum region.

Conversely, monitoring the inner branch behavior for a soil environment might become challenging due to the very same foundations, especially in terms of small magnitude and phase impedance fluctuations at consistently high frequencies for allowing it. Such a specific topic was not hereby profoundly focused but should be if such a fulfillment ever becomes the primary measurement to be investigated in the future.

### 3.3. Non-Ideal Effects

Any change in the humidity of the terrain, such as, for instance, that produced by rain, reaching the sensitive volume above the electrodes would be detected by the sensor and would produce a false leak alarm. In order to avoid false alarms, we foresee two strategies. One is the combination of local impedance data with meteorological information or with a global surface humidity detector. This would allow for rejecting a false trigger in the presence of an increase in humidity due to rainfall. The second strategy, here experimentally demonstrated and definitely more effective, is the adoption of a differential sensing approach. In this section, we first study the impact on non-ideal effects, such as temperature and soil morphology, resulting in artefacts in the sensed impedance and then validate a differential sensing scheme.

#### 3.3.1. Temperature

**Figure 13:**
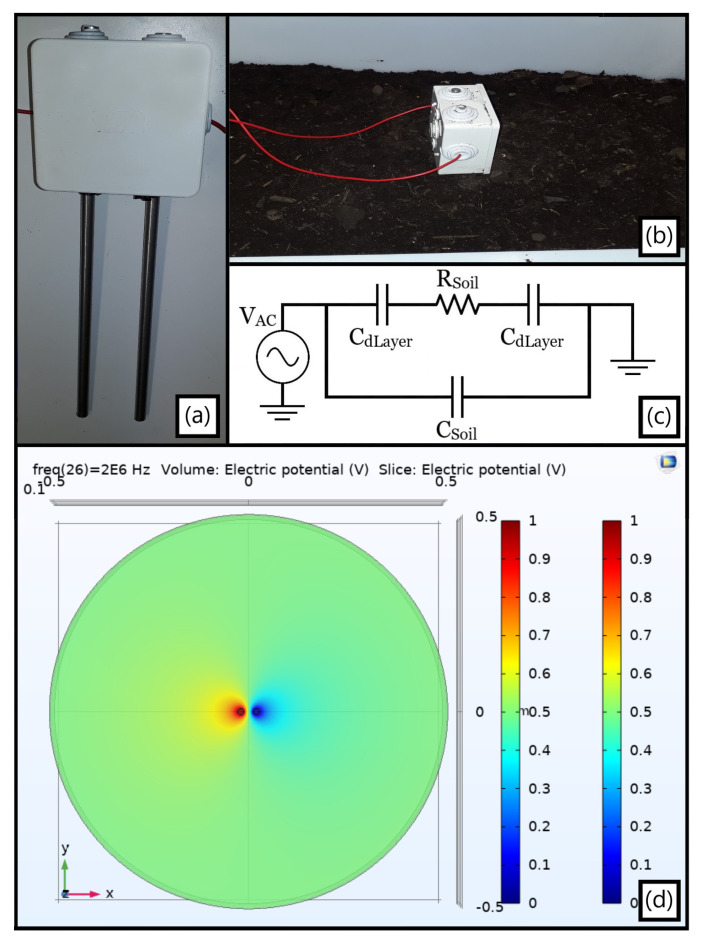
(**a**) Moisture probe with two parallel inox rods; (**b**) Vertically positioned device inside the plastic box soil; (**c**) Simplified electrical equivalent circuit sensed by the equipment; (**d**) Three-dimensional F.E.M. outcomes yielded at 2 MHz and viewed at the xy plane for the single metallic rods pair in a wide 50 cm-radius homogeneous terrain with no insulating perimeter.

Temperature affects the value of water conductivity by from 7.5% to 2.5% per °C, and this effect was experimentally characterized. For analyzing the pipe-encircling environmental electrical conductivity as a function of temperature, a low-cost moisture probe was built as in Figure 13a. In specific, two 4 mm-radius steel bars were cut to a 20 cm length and positioned in parallel to each other through a tiny plastic case, which holds the system parallelism and separates their centroids by a 4 cm distance. The impedance measurement itself was set by welding a wire to each metallic rod on one end and to a BNC on the other, allowing then the experimental gauging by the LCR utilized so far to be performed.

The subsequently extracted data was gathered not only by inserting the probe vertically into another rectangular plastic box containing the soil (Figure 13b), but also by positioning it vertically and horizontally as well as rotating it to be orthogonal first to the longest box side and afterwards to its shortest one. As forecasted, an inherent geometrical dependence analogous to the minimal ground radius principle focused on in Section 3.1.2 was retestified once distinct yet almost in-phase absolute impedances were obtained. However, the system was able, in all instances, to be reasonably approximated by the simplified equivalent circuit from Figure 13c, exhibiting a |Z(jω)| plateau at intermediate frequencies associated to R_Soil_, ergo related to σ_Soil_, and a capacitive comportment otherwise.

A prime foundation for such a spurious effect presumably relies on the electric field spatial dispersion, as in the F.E.M. simulation from Figure 13d: albeit the probe’s inner hemi circles electric field resembles that of equivalent parallel plates, the outer ones disseminate much farther, hence being potentially confined by the insulating plastic walls. Nonetheless, solely altering temperature while ensuring a stationary terrain geometry and probe position was later proven sufficient for estimating the ∂σ_Soil_(T)/∂T targeted behavior.

**Figure 14:**
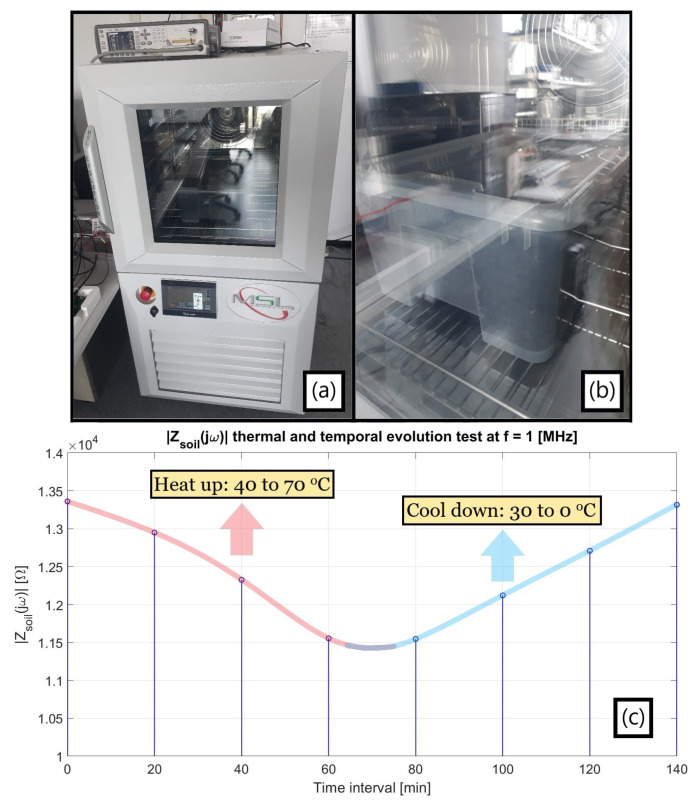
(**a**) Thermal chamber apparatus with LCR impedance analyzer standing on top of it; (**b**) Detail on small plastic packaging enfolding the horizontally buried low-cost moisture probe, with a red wiring bonding the latter to the external LCR; (**c**) Thermal and temporal evolution of the terrain |Z_Soil_(jω)| at 1 MHz, empirically advocating for the positive ∂σ_Soil_(T)/∂T µS/cm·°C.

A thermal chamber was utilized (Figure 14a) to heat a ground sample within a small box, yet this was able to adequately fit the moisture probe in it (Figure 14b) for keeping track of the terrain impedance. The established procedure for this novel environment was to first set the apparatus to a target temperature, assess the probe after a 20 min period as an endeavor for achieving a thermal homogeneity within the investigated soil, and reiterate such cycle for a few times.

As presented in Figure 14c, a heating-up process (40 to 70 °C) preceded the immediate cooling-down one (30 to 0 °C) for circumventing eventual misleading drift effects, consistently maintaining the aforesaid impedance spectrum time sampling interval as well. The highlighted frequency was 1 MHz from being in the same order of magnitude as that potentially targeted for the water loss sensing, notwithstanding the fact that a capacitive behavior dominates the system at this range as later detailed. In any case, the plot still successfully endorses the primary argument of a foretold positive ∂σ_Soil_(T)/∂T since the terrain resistance in parallel to its quasi-constant capacitance decreases (increases) due to a greater (lesser) σ_Soil_ at higher (lower) temperatures, and so does the entire |Z_Soil_(jω)|.

**Figure 15:**
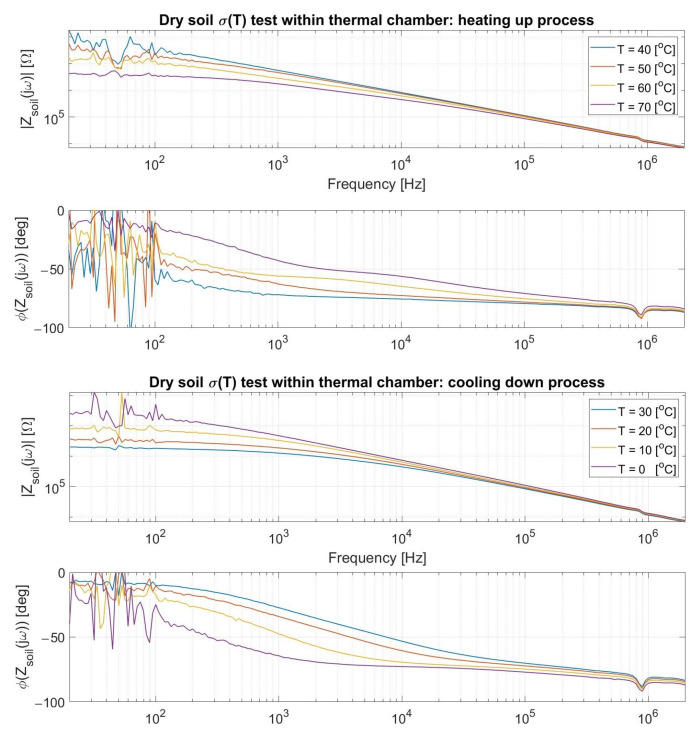
Yielded spectra for a set of temperatures via the current thermal chamber procedure.

A noteworthy aspect in the above plot regards how the soil core encompassing the probe does not precisely reproduce the chamber temperature, but merely mirrors its temporal oscillation. Specifically, the abrupt climate shift from 70 to 30 °C is followed by an impedance preservation likely resulting from a conserved heat at the inner terrain portion until its cooling down is de facto triggered, which is a recurrent feature throughout the full spectra presented in Figure 15 for these values. Therefore, the ground apparently tends to better accompany inertial thermal transitions rather than sudden opposing ones.

Moreover, the proximity between |Z(jω)| at t = 0 and 140 min consists perhaps of a coincidence from probably reaching inner soil temperatures close to each other although at pretty distinct chamber ones. Certainly, the last gauged soil impedance should considerably surpass the first one measured at 40 °C if it were retained at 0 °C for longer than analyzed. Finally, it is emphasized that the attained spectra must consider electrical element magnitudes virtually larger than the real ones due to the plastic box walls almost adjacent to the probe rods, even though no nefarious impact is, in principle, entailed from it when analyzing their modification exclusively from temperature as abovementioned. 

As witnessed in the antecedent charts, a resistive plateau is followed by a dominating capacitive demeanor in compliance to the hypothesized equivalent circuit from Figure 13c, whose description followed the preliminary experiments developed. In addition, the access capacitances from the ionic double layer encircling the metallic probes surfaces (C_dL_, which might be better modeled by a Constant Phase Element) can also be inspected from the non-purely resistive phase at the quasi-DC range due to a small zero singularity (f_Z,CdL_~1/2πC_dL_R_Soil_) with a virtually high R_Soil_. Lastly, the primary observed pole (f_P_~1/2πC_Soil_R_Soil_) decreases when R_Soil_ rises in colder environments and increases in hotter ones, i.e., σ_Soil_ becomes poorer and greater, respectively, for an a priori fairly steady parallel C_Soil_.

#### 3.3.2. Soil Morphology

**Figure 16:**
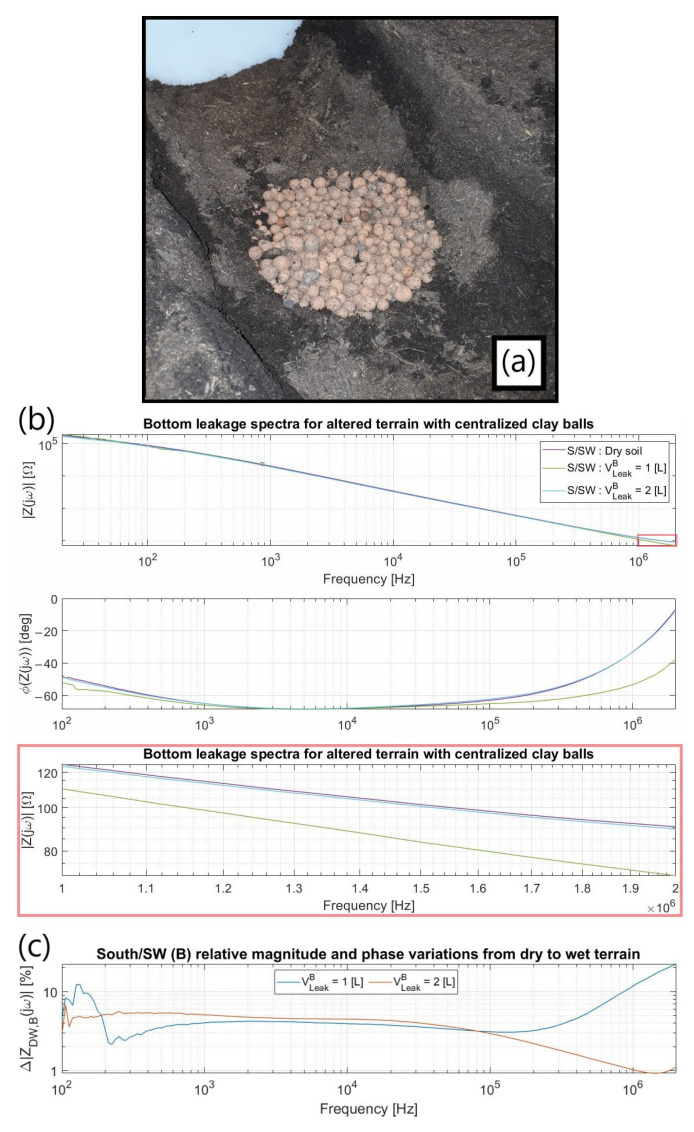
(**a**) Setup after bottom leakage (V^B^_Leak_) ceased; (**b**) Attained spectra, S/SW being the electrode “triplet” used (concept explained in Section 3.4). The zoom in red highlights the impedance variation at the zone where R_Soil_ starts to take over; (**c**) Idem, but normalized by a dry soil baseline.

A centralized hole about 10 cm wide in diameter and 20 cm in depth, entirely filled with medium-sized impermeable clay balls (Figure 16a), simulated the terrain morphological alteration, hence allowing the concentric pumped leakage to flow away from the electrodes much faster than for a regular commercial porous soil. As inferred by the measured spectra (Figure 16b), the water likely accumulates close to the electrodes at first but rapidly moves away from them for greater pumped volumes. Although monitoring R_Soil_ at the MHz range becomes a burden in this situation, smaller frequencies still demonstrated increasingly leak-dependent impedance fluctuations (Figure 16c), which might then be used as an alternative to handle the leakage detection capability for such a worst-case scenario. 

### 3.4. Differential Measurements 

A feasible solution to avoid false positive detections triggered by multiple sources producing a Δ|Z(jω)| > 0, is the adoption of a differential approach. Individual electrodes are biased sequentially and the changes of the impedance values are compared between neighbor electrodes. Consequently, not only can common-mode humidity and other spurious fluctuations be suppressed, but the leak profile can also be estimated. In principle, an angular sector impedance readout can be performed to prioritize a particular cardinal direction analysis by adequately leaving each electrode unbiased or connected via analog multiplexers at either V_DD_ or virtual ground (V.G.). More specifically, each metal strip can be individually biased at V_DD_ in a sequence with at least its two adjacent electrodes at the V.G., whence the output current flows, so to better discretize and estimate the real leakage φ-profile relying on a Δ|Z(jω)| polar plot. Nonetheless, only four of them were selected as such, since mitigating power consumption is also an indispensable goal.

**Figure 17:**
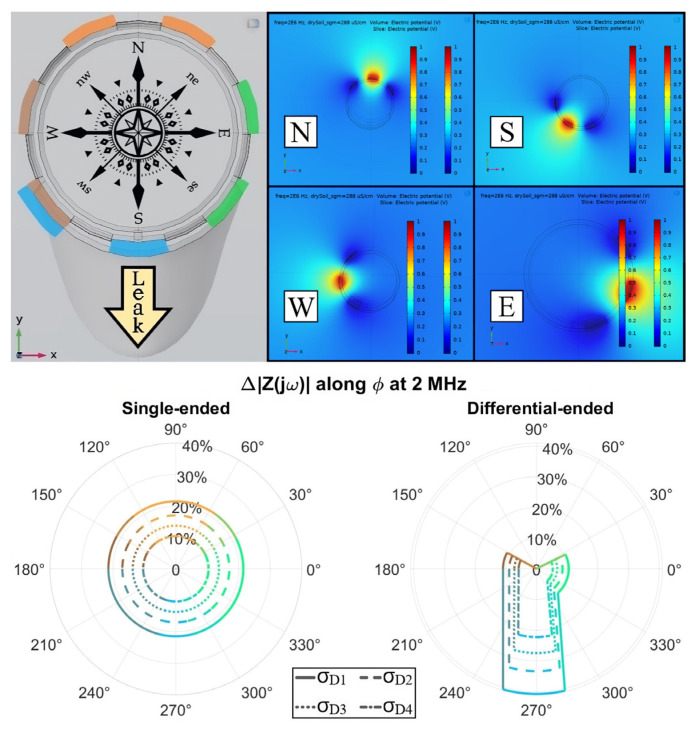
(**Top**) Sensor frontal view with electrodes subdivided into triplets and their respective F.E.M. simulations, unused metal strips being absent in the latter; (**Bottom**) Both single-ended and angular differential emulated readouts are shown following the predefined color pattern, with four distinct electrical “dry soil” conductivities (σ_D4_ > σ_D3_ > σ_D2_ > σ_D1_) mimicking a common-mode humidity homogeneously scattered within the terrain and degrading the system’s sensitivity to a leak.

As exhibited in Figure 17, an ensemble of F.E.M. simulations at 2 MHz was first evaluated to endorse such a proposition, hypothesizing a homogeneous terrain encircling the detector with a conductivity spanning equally spaced intervals towards that of the leak zone itself. Therefore, the σ_D1–_σ_D4_ increasing values imposed for the confined soil portion not straightforwardly impacted by the water loss, henceforth denominated “dry soil”, were 180, 216, 252, and 288 μS/cm, respectively, σ_Leak_ = 500 μS/cm being within the 2 L Gaussian-alike domain when existent. Such an F.E.M. model had to exclude electrodes whose potential would be floating because no convergence was obtained otherwise.

Moreover, the alternative condition with all of them connected to the V.G. except that biased at V_DD_ was scrutinized as well. As an expected consequence from the electromagnetic fields’ spatial dispersion for the studied geometry, no relevant difference was testified between the Δ|Z(jω)| for the antecedent option and this other one. The former is hereby displayed due to being closer to that later verified empirically, albeit the latter would ensure a better robustness to noise in a real scenario, and hence a greater SNR.

Concerning the cardinal diagram, one specific color was attributed to the gaps encompassed by an electrode triplet, each of which was nominated by the nearest pointed-out directions when the reference basis is aligned to the defined compass rose, namely North (N), South (S), West (W), and East (E). A slight intrinsic asymmetry emerged within such analysis once seven metal strips were utilized instead of eight, which would detach the overlapping gap divvied up by W and S. The subsequent polar plots preserved such oriented colors, however, averaging their RGB coordinates along angular sectors shared by distinct triplets. Similarly, the radii values themselves representing Δ|Z(jω)| were linearly interpolated for adjacent triplet results whose relative difference was inferior to 20%. Contrariwise, straight lines were imposed to highlight the contrast between parched and soaked soil regions. 

In Table 5, the differential-ended dry soil baseline (BL) for no leak present was computed only once and reused for all cardinal analyses due to its symmetry along the several triplets. At the 2 MHz target frequency, there is a predominant impact of the external environment’s resistive behavior on |Z(jω)|, which becomes hyperbolically proportional to σ_env_ as a result. Furthermore, since the current flow faces a narrower equivalent surface from single-ended to differential measurements, the dry BL at Table 5 increased roughly by a factor 7/3, i.e., the ratio between the number of electrodes utilized. In both biasing schemes, the ∆|Z(jω)| sensitivity peaks occurred when the driest initial baseline (σ_D1_) was imposed because ∂|Z(jω)|/∂σ has a magnitude proportional to 1/σ^2^, being then larger for smaller conductivities.

Regarding the single- and differential-ended leak detection capability comparison, ∆|Z(jω)| was essentially doubled for the South triplet since it is that much better at focusing the infiltration zone. This value ought to be even greater if the S-triplet were entirely enfolded by such an emulated domain, a non-occurrence due to the above-mentioned asymmetry inherent to the sensor. Moreover, a steady and ideally null impedance oscillation was sensed by the N-triplet, although the forthcoming empirical tests suggest the existence of some background sensitivity even for electrodes not directly affected by the water loss. Nevertheless, this circumstance does not jeopardize the leakage detection as better exemplified in the following. Finally, the zero-detection limit in the two cases is forecasted to happen when both conductivities from the common-mode humidity and the confined infiltration tend to coincide.

**Figure 18:**
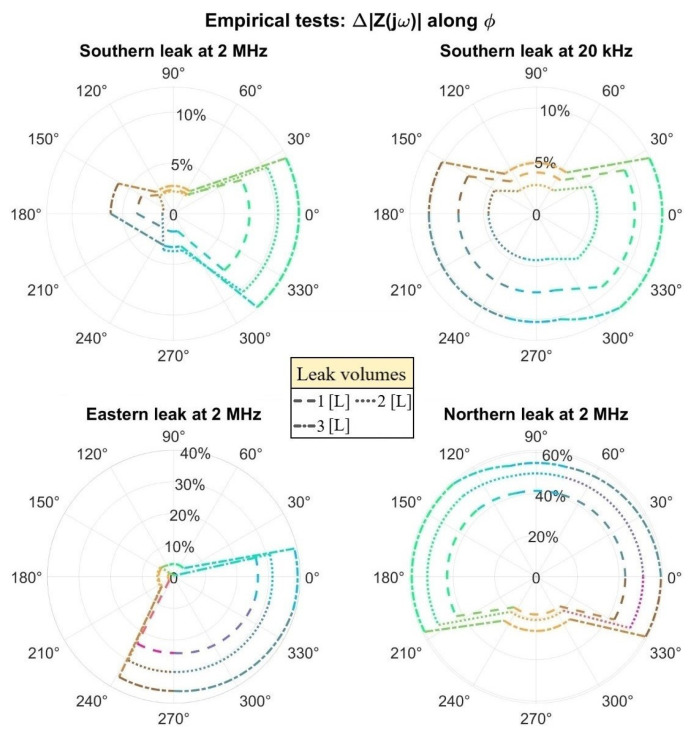
Polar plots attained for ∆|Z(jω)|, evaluated throughout the φ cylindrical coordinate for three distinctly localized leakage sources. In each of them, the four cardinally oriented triplet gauged values are interpolated along intermediate shared regions when sufficiently contiguous or depicted by steep lines otherwise, being represented by the vicinities’ averaged colors in both cases.

As for the real case tests, the three primary leak scenarios were sequentially assayed as presented in Figure 18, imposing an increasing water volume in all of them, fleeing at first from the South. The system was further rotated anticlockwise to also emulate the Eastern and Northern losses, maintaining, however, the identical denomination and colors from Figure 17 for the triplets. In the latter plots, pinkish curves were inserted to coherently replace a few outlier measurements. A subdivision into four triplets connected to biasing signals was established with the remaining unused electrodes left floating as simulated by the F.E.M. model, allowing, in any case, a consistent estimation of the water seepage profile into the soil.

A foremost argument to enlighten the preceding outcomes is the spatial domain within which the metal strips’ electromagnetic field is mainly concentrated, which limits the sensor detection range to a maximum radius of about 15 cm above the pipe surface. For instance, the Southern plot indicates an actual propensity for water to flow straight to the bottom with a slight aqueous clustering in the East on the contrary of ideally building up near the bottom electrodes as assumed by the CAD model, an occurrence unnoticeable through the single-ended scheme. Therefore, the real sensitivity is reduced compared to the digital prediction, even though both the leakage existence and its profile can still be consistently unraveled. 

Founded on a similar reasoning, progressively increasing absolute impedance variations were achieved when the disseminating water directly covered more electrodes in place of flowing radially away from them. As can be witnessed on Figure 18, the Eastern leak variations were considerably enlarged with respect to the Southern ones once the loss spilled over about half of the S- and W-triplets from Figure 17. Analogously, when all individual strips within the triplets were promptly encompassed by the aqueous dispersion as in the Northern case, a ∆|Z(jω)| peak variation of 60% was testified. Interestingly, the triplet on top seems to reach a detection limit in this condition likely due to gravity: the fluid tends to preferably gather around the lateral electrodes for a 3 L volume, ergo being better sensed by them. 

Despite defining 2 MHz as the frequency of interest, other ones could also conveniently provide useful information about the leak. Specifically, a recurrent ∆|Z(jω)| > 0 did systematically occur at the CPE portion from all evaluated spectra, e.g., at the 20 kHz vicinities as displayed in Figure 18. However, reducing the readout frequency compromises the impedance SNR, though diminishing electronics costs, since a smaller readout current is obtained for a constant applied voltage, i.e., |Z(jω)| has ∂|Z(jω)|/∂ω < 0 for the studied range. In any case, such Δ|ε_r,CPE_| disturbance remains as a potentially valid cross-checking procedure for the Δσ_Soil_ main tracking goal as in the Southern loss comparison in Figure 18. In addition, both parameters entailed non-negligible ∆|Z(jω)| values at the latter N-triplet, which should ideally not have been affected. Hence, adopting a minimal fluctuation to be ratified as relevant might also become crucial in real applications to overcome such background detections.

Finally, false triggering events can be minimized within the differential perspective by substituting the detection protocol itself; instead of tracking the individually gauged ∆|Z(jω)|, the relative variation among adjacent triples can be assessed by the factor

(4) $$S_{D}=\left|1-\frac{{∆}_{1}\left|Z\left(j\omega \right)\right|}{{∆}_{2}\left|Z\left(j\omega \right)\right|}\right|$$

In particular, the central unit can be informed about a water loss occurrence exclusively if S_D_ overcomes a certain predefined threshold (T), a coefficient not to be misread with the absolute percentages per se exhibited on the polar plots from Figure 18. For a small preset T > 0, e.g., T = 0.2 as hereby adopted, such an S_D_ > T condition can be approximately rewritten as ∆_2_|Z(jω)| being respectively greater or minor than (1 ± T)*·*∆_1_|Z(jω)| and graphically expressed via the straight lines connecting distinct sensitivities on the plots. For a thorough consistency, S_D_ can be computed as well between diametrically opposed triplets: relying on Table 4, S_D_(W,E) → 0 whereas S_D_(N,S) >> T. In principle, these indicators denote a leakage flowing either from the pipe bottom portion, as in Figure 17, or symmetrically from its top.

The same method here discussed could be applied for sequentially embedded electrodes throughout the tube length. In specific, the S_D_ factor can be determined among outcomes from a constant cardinal direction but distinct z-axis coordinates to similarly predict the water loss along the conduit length. Therefore, once both angular and longitudinal indicators have been persistently triggered by continuous assessments and in possibly more than a single frequency, substantial evidence will have been collected for the central unit to attest an ongoing leakage within the water distribution network.

## 4. Discussion

The main challenge of a leak sensor aiming at monitoring a large network is to combine a long length of monitored pipe (L) with a small minimum detectable volume (V). Considering, in particular, the random nature of the leakage and the unpredictable shape and orientation, the technique should be able to monitor the whole pipe surface. We have optimized the distance (G) and the width (W) of the electrodes in order to maximize both the pipe surface coverage and sensitivity.

In order to situate the results here reported with respect to the state of the art, we compare them with other techniques sensitive to small leaks (i.e., below 10 L), namely optical fibers [23], smart flow meters [24], and pressure sensing [25]. As summarized in Table 6, impedance allows for monitoring of a long segment of pipe (up to 6 m), with a volume resolution of 0.5 L and a fast response time (~1 s). When combining these parameters into a single figure of merit (FoM) highlighting the ratio between the monitored length and the minimum detectable volume, our technique ranks among the top ones.

Another key aspect of a buried sensor is its stability and reliability. Since the electrodes are insulated, being sandwiched between the plastic pipe and the Kapton passivation layer, a direct contact with water is excluded. No degradation nor drift were observed in our tests, which were running for weeks (temperature being the main source of drift, rejected by differential configurations). The most critical aspect in terms of reliability in the field is expected to be the electrical connection between the pipe strip electrodes and the readout instrument. In the experiments here reported, wires were soldered on copper pads exposed through the Kapton passivation. A possible solution for industrial deployment could be a ring with metallic contacts clamped and fastened around the pipe in a region where copper is exposed. 

The signal-to-noise ratio was set, in our laboratory experiments, by the measurement instrument. In fact, the electrodes are connected to two low-impedance points: the forcing potential at one terminal and the input virtual ground at the other one. Especially when deploying long electrodes, the effect of electromagnetic pickup should be carefully assessed in the field. We expect that the water running in the pipe and the earth surrounding it should act as shields.

## 5. Conclusions

We presented a detailed simulation analysis and experimental characterization of an impedance sensor able to early detect water leaks as small as 1 L. Considering that minor leaks have flow rates in the other of ~liter/min, the presence of a leak could be detected in a few minutes. Experiments were performed with a length of electrodes of 45 cm. However, simulations, once optimized to fit experimental data, show that impedance variations of a few % of ~50 Ω are achievable with the same volume of leak for electrodes up to 6 m.

Differential configurations (either among electrode pairs within the same sector or among different pipe sectors) represent the most robust way to reject spurious impedance changes due to environmental effects, such as rainfall that increases the ground humidity and is thus common to multiple sensing regions, such as humidity and temperature, that were here characterized. 

Different avenues of improvement can be envisioned. In order to improve sensitivity, one action could be to coat electrodes with a porous material, able to retain water close to the electrodes and, thus, increase the impedance change. Another important perspective is the application of machine learning, currently adopted for leakage prediction and identification with traditional sensors [26], to the classification of impedance data in order to reduce leak identification false alarms and potentially automatically correlate several sources of information.

Finally, the next development step will be the design of a custom electronic unit able to sense impedance variations with the proper sensitivity (a few %) and in the frequency range of 1–10 MHz with phase-sensitive capabilities. In fact, although our measurement instrument is limited at 2 MHz, simulations indicate an optimal sensing frequency in the middle of that decade. The system should be compact and low-power in order to be hosted in a wireless sensing node, along with other Internet-of-Things technologies, for smart and real-time water distribution monitoring.

## Figures and Tables

**Table 1:** Parameterization example focusing on the Gaussian-alike f(x,y,z) top surface equations.

$X\left(\theta_{G},z_{G}\right)=cos\left(\theta_{G}\right)\cdot \left\{\rho_{0,G}+A_{G}\cdot \exp\left\{-\left[\frac{{\left(z_{G}-z_{0,G}\right)}^{2}}{2\;\cdot \;\sigma_{z_{G}}^{2}}+\frac{{\left(\theta_{G}-\theta_{0,hole}\right)}^{2}}{2\;\cdot \;\sigma_{z_{G}}^{2}}\right]\right\}\right\}$
$Y\left(\theta_{G},z_{G}\right)=sin\left(\theta_{G}\right)\cdot \left\{\rho_{0,G}+A_{G}\cdot \exp\left\{-\left[\frac{{\left(z_{G}-z_{0,G}\right)}^{2}}{2\;\cdot \;\sigma_{z_{G}}^{2}}+\frac{{\left(\theta_{G}-\theta_{0,hole}\right)}^{2}}{2\;\cdot \;\sigma_{z_{G}}^{2}}\right]\right\}\right\}$
*Z (* $z_{G}$ *) = z_G_*

**Table 2:** Z_Soil_(jω) [Ω] at 1 kHz for variables *h_Leak_*(*V_Leak_*) and *σ_Leak_* (in µS/cm), starting from a confined leak zone baseline (BL) conductivity equal to the adjacent ground’s one, i.e., fully dry soil. The background colors act as a reminder for the arid soil (yellowish) gradually becoming wet (blueish).

Z_Soil_ @1kHz (σ_L_, h_L_)	V_Leak_ = 0.25 [L] (h_L_~7.3 [cm])	V_Leak_ = 0.5 [L] (h_L_~12.4 [cm])	V_Leak_ = 0.75 [L] (h_L_~16.5 [cm])	V_Leak_ = 1.0 [L] (h_L_~20 [cm])
1.0 (BL)	20,426 ∠ −0.82°	20,426 ∠ −0.82°	20,426 ∠ −0.82°	20,426 ∠ −0.82°
25.0	12,755 ∠ −0.50°	12,715 ∠ −0.50°	12,706 ∠ −0.50°	12,701 ∠ −0.50°
50.0	9293 ∠ −0.37°	9268 ∠ −0.36°	9264 ∠ −0.36°	9260 ∠ −0.36°
100.0	6030 ∠ −0.24°	6016 ∠ −0.24°	6015 ∠ −0.24°	6013 ∠ −0.24°
150.0	4463 ∠ −0.18°	4455 ∠ −0.18°	4454 ∠ −0.18°	4453 ∠ −0.18°
200.0	3544 ∠ −0.14°	3537 ∠ −0.14°	3537 ∠ −0.14°	3536 ∠ −0.14°
300.0	2509 ∠ −0.10°	2505 ∠ −0.10°	2505 ∠ −0.10°	2504 ∠ −0.10°
400.0	1942 ∠ −0.08°	1939 ∠ −0.08°	1939 ∠ −0.08°	1938 ∠ −0.08°
500.0	1584 ∠ −0.06°	1582 ∠ −0.06°	1582 ∠ −0.06°	1581 ∠ −0.06°

**Table 3:** Z_Soil_(jω) [Ω] and Δ|Z_DW_(jω)| [%] at 2 MHz as a function of the terrain radius (*ρ_Soil_*) and the water leak volume (*V_Leak_*) departing from a dry soil baseline (BL). The background colors on top act as a reminder for the arid soil (yellowish) gradually becoming wet (blueish), whereas the ones on the left stand for the incorrect setup (light red) vs. the corrected one (light green).

Z_Soil_(jω)	Dry (BL)	0.5 [L]	1.0 [L]	1.5 [L]	2.0 [L]	3.0 [L]	4.0 [L]
Δ|Z_DW_(jω)|							
5 [cm]	129.6 ∠−24.4°	117.9 ∠−24.5°	108.8 ∠−25.9°	99.9 ∠−27.0°	88.3 ∠−29.0°	--	--
	0%	9.0%	16.1%	22.9%	31.9%		
17 [cm]	55.8 ∠−13.6°	--	50.5 ∠−12.5°	--	48.5 ∠−11.8°	47.5 ∠−11.1°	47.0 ∠−10.7°
	0%		9.5%		13.1%	14.9%	15.8%

**Table 4:** Obtained parameters once refined until reaching convergence between measured and simulated spectra from dry to wet (2 L leak) situations for shallower and thicker terrain setups. In the latter, the ionic double layer and the polyimide one were unified for a better consistency. The background colors on the table act as a reinforcement for the arid soil (yellowish) counterposing the wet one (blue), whereas the incorrect setup radius (light red) opposes the corrected one (light green).

Materials	ρ_Soil_ = 5 [cm]		ρ_Soil_ =17 [cm]	
	Dry	Wet	Dry	Wet
Ground **^(1)^**	σ = 45 ε_r_ = 7.5	σ = 400 ε_r_ = 60	σ = 180 ε_r_ = 2.5	σ ∊ [300, 400] ε_r_ = 60
Ionic double Layer **^(2)^**	σ = 115 ε = 0.35	σ = 400 ε = 0.5	σ = 20 ε_r_ = α ε_Kp_(jω)^−0.17^, α = 2, ε_Kp_ = 3	σ = 50 ε_r_ = α ε_Kp_(jω)^−0.17^ α > 2 (α ∊ ℝ), ε_Kp_ = 3
Kapton*^®^* **^(2)^**	σ = 115 ε_r_ = 3 **^(3)^**	σ = 115 ε_r_ = 3		

**^(1)^** σ in µS/cm; **^(2)^** σ in pS/cm; **^(3)^** Average permittivity value for commercial tapes.

**Table 5:** Absolute impedances obtained from F.E.M. numerical simulations at 2 MHz for both single-ended (S.E.) and differential-ended (D.E.) biasing schemes as depicted in Figure 17. The latter’s color pattern is followed for the D.E., whereas the S.E. color was added for better readability only.

|Z(jω)|[Ω]	σ_D1_ (180 [μS/cm])	σ_D2_ (216 [μS/cm])	σ_D3_ (252 [μS/cm])	σ_D4_ (288 [μS/cm])
S.E. Dry BL	38.68	32.51	28.11	24.81
S.E. Wet	30.38	26.98	24.33	22.22
D.E. Dry BL	104.50	87.80	75.88	66.95
D.E. (N) Wet	104.49	87.79	75.88	66.95
D.E. (W) Wet	93.07	79.99	70.41	63.08
D.E. (S) Wet	62.77	58.55	55.06	52.10
D.E. (E) Wet	93.50	80.37	70.76	63.40

**Table 6:** Comparison of the sensing performance of different detection techniques with sensitivity to small leaks.

Technology	L [m]	V [L]	t [s]	FoM *	Ref.
Impedance	6	0.5	1	12	This work
Optical Fiber	0.02	0.005	600	0.007	[23]
Smart Meter	1	0.008	10	12.8	[24]
Pressure	100	7	10	1.4	[25]

* The Figure of Merit, to be maximized, is FoM = L/(V·t), where L is the monitored length, V the minimum detectable volume, and t the response time.

## Data Availability

Data are available upon reasonable request.

## References

[1] Hoekstra A.Y., Mekonnen M.M. (2012). The Water Footprint of Humanity. Proc. Natl. Acad. Sci. USA.

[2] El-Zahab S., Zayed T. (2019). Leak Detection in Water Distribution Networks: An Introductory Overview. Smart Water.

[3] Islam M.R., Azam S., Shanmugam B., Mathur D. (2022). A Review on Current Technologies and Future Direction of Water Leakage Detection in Water Distribution Network. IEEE Access.

[4] Pudar R.S., Liggett J.A. (1992). Leaks in Pipe Networks. J. Hydraul. Eng..

[5] Levinas D., Perelman G., Ostfeld A. (2021). Water Leak Localization Using High-Resolution Pressure Sensors. Water.

[6] Bach P.M., Kodikara J.K. (2017). Reliability of Infrared Thermography in Detecting Leaks in Buried Water Reticulation Pipes. IEEE J. Sel. Top. Appl. Earth Obs. Remote Sens..

[7] Sneddon K.W., Olhoeft G.R., Powers M.H. Determlning And Mapplng Dnapl Saturation Values From Nonlnvasive Gpr Measurements. Proceedings of the 13th EEGS Symposium on the Application of Geophysics to Engineering and Environmental Problems; European Association of Geoscientists & Engineers.

[8] Okosun F., Cahill P., Hazra B., Pakrashi V. (2019). Vibration-Based Leak Detection and Monitoring of Water Pipes Using Output-Only Piezoelectric Sensors. Eur. Phys. J. Spec. Top..

[9] Gao Y., Brennan M.J., Joseph P.F., Muggleton J.M., Hunaidi O. (2004). A Model of the Correlation Function of Leak Noise in Buried Plastic Pipes. J. Sound Vib..

[10] Xu J., Chai K.T.-C., Wu G., Han B., Wai E.L.-C., Li W., Yeo J., Nijhof E., Gu Y. (2019). Low-Cost, Tiny-Sized MEMS Hydrophone Sensor for Water Pipeline Leak Detection. IEEE Trans. Ind. Electron..

[11] Mohd Ismail M.I., Dziyauddin R.A., Ahmad Salleh N.A., Muhammad-Sukki F., Aini Bani N., Mohd Izhar M.A., Latiff L.A. (2019). A Review of Vibration Detection Methods Using Accelerometer Sensors for Water Pipeline Leakage. IEEE Access.

[12] Ren L., Jiang T., Jia Z., Li D., Yuan C., Li H. (2018). Pipeline Corrosion and Leakage Monitoring Based on the Distributed Optical Fiber Sensing Technology. Measurement.

[13] Ibrahim K., Tariq S., Bakhtawar B., Zayed T. (2021). Application of Fiber Optics in Water Distribution Networks for Leak Detection and Localization: A Mixed Methodology-Based Review. H2Open J..

[14] Cataldo A., Persico R., Leucci G., De Benedetto E., Cannazza G., Matera L., De Giorgi L. (2014). Time Domain Reflectometry, Ground Penetrating Radar and Electrical Resistivity Tomography: A Comparative Analysis of Alternative Approaches for Leak Detection in Underground Pipes. NDT E Int..

[15] Jordana J., Gasulla M., Pallàs-Areny R. (2001). Electrical Resistance Tomography to Detect Leaks from Buried Pipes. Meas. Sci. Technol..

[16] D’Adda I., Battaglin G., Carminati M. (2021). A Low-Cost Flexible Pipe Sheath for Multi-Parameter Monitoring of Water Distribution. Proceedings of the 2021 IEEE International Symposium on Circuits and Systems (ISCAS).

[17] Luo B., Wang T., Zhang F., Lin Y., Zheng C., Chen S. (2021). Interdigital Capacitive Sensor for Cable Insulation Defect Detection: Three-Dimensional Modeling, Design, and Experimental Test. J. Sens..

[18] Quan J., Shi X., Kim K.N., Oh D.S., Chua B. (2023). Electrically Functional Water Sensing Duct Tape Suitable for Detection of Indoor Pinhole Leakages with High Spatial and Temporal Resolution. Sens. Actuators A Phys..

[19] Carminati M., Ferrari G., Vahey M.D., Voldman J., Sampietro M. (2017). Miniaturized Impedance Flow Cytometer: Design Rules and Integrated Readout. IEEE Trans. Biomed. Circuits Syst..

[20] Carminati M., Mezzera L., Turolla A., Pani G., Tizzoni M., Di Mauro M., Antonelli M. (2019). Flexible Impedance Sensor for In-Line Monitoring of Water and Beverages. Proceedings of the 2019 IEEE International Symposium on Circuits and Systems (ISCAS).

[21] Riboldi C., Castillo D.A.C., Crafa D.M., Carminati M. (2023). Contactless Sensing of Water Properties for Smart Monitoring of Pipelines. Sensors.

[22] Demierre N., Braschler T., Linderholm P., Seger U., van Lintel H., Renaud P. (2007). Characterization and Optimization of Liquid Electrodes for Lateral Dielectrophoresis. Lab Chip.

[23] Cho T.-S., Choi K.-S., Seo D.-C., Kwon I.-B., Lee J.-R. (2012). Novel Fiber Optic Sensor Probe with a Pair of Highly Reflected Connectors and a Vessel of Water Absorption Material for Water Leak Detection. Sensors.

[24] Pietrosanto A., Carratù M., Liguori C. (2021). Sensitivity of Water Meters to Small Leakage. Measurement.

[25] Casillas Ponce M.V., Garza Castañón L.E., Cayuela V.P. (2014). Model-Based Leak Detection and Location in Water Distribution Networks Considering an Extended-Horizon Analysis of Pressure Sensitivities. J. Hydroinform..

[26] Fan X., Zhang X., Yu X. (2021). Machine Learning Model and Strategy for Fast and Accurate Detection of Leaks in Water Supply Network. J. Infrastruct. Preserv. Resil..

